# Changes in Plasma Soluble Receptor for Advanced Glycation End-Products Are Associated with Survival in Patients with Acute Respiratory Distress Syndrome

**DOI:** 10.3390/jcm10102076

**Published:** 2021-05-12

**Authors:** Matthieu Jabaudon, Bruno Pereira, Erwan Laroche, Laurence Roszyk, Raiko Blondonnet, Jules Audard, Thomas Godet, Emmanuel Futier, Jean-Etienne Bazin, Vincent Sapin, Julie A. Bastarache, Lorraine B. Ware, Jean-Michel Constantin, on behalf of the LIVE Study Group and the AZUREA Network

**Affiliations:** 1 Department of Perioperative Medicine, CHU Clermont-Ferrand, 63000 Clermont-Ferrand, France; elaroche@chu-clermontferrand.fr (E.L.); rblondonnet@chu-clermontferrand.fr (R.B.); jaudard@chu-clermontferrand.fr (J.A.); tgodet@chu-clermontferrand.fr (T.G.); efutier@chu-clermontferrand.fr (E.F.); jebazin@chu-clermontferrand.fr (J.-E.B.); 2 GReD, CNRS, INSERM, Université Clermont Auvergne, 63000 Clermont-Ferrand, France; lroszyk@chu-clermontferrand.fr (L.R.); vsapin@chu-clermontferrand.fr (V.S.); 3 Division of Allergy, Pulmonary, and Critical Care Medicine, Department of Medicine, Vanderbilt University Medical Center, Nashville, TN 37232, USA; julie.bastarache@vumc.org (J.A.B.); lorraine.ware@vumc.org (L.B.W.); 4 Biostatistics and Data Management Unit, Department of Clinical Research and Innovation (DRCI), CHU Clermont-Ferrand, 63000 Clermont-Ferrand, France; bpereira@chu-clermontferrand.fr; 5 Department of Biochemistry and Molecular Genetics, CHU Clermont-Ferrand, 63000 Clermont-Ferrand, France; 6 Department of Cell and Developmental Biology, Vanderbilt University, Nashville, TN 37232, USA; 7 Department of Pathology, Microbiology, and Immunology, Vanderbilt University Medical Center, Nashville, TN 37232, USA; 8 Department of Anaesthesia and Critical Care, DREAM, Sorbonne Université, Hôpital Pitié-Salpêtrière, Assistance Publique-Hôpitaux de Paris, 75013 Paris, France; jean-michel.constantin@aphp.fr

**Keywords:** acute respiratory distress syndrome, soluble RAGE, biomarker, joint modeling, therapeutic response, mechanical ventilation

## Abstract

The plasma soluble receptor for advanced glycation end-products (sRAGE) is a marker of lung epithelial injury with prognostic value when measured at baseline in acute respiratory distress syndrome (ARDS). However, whether changes in plasma sRAGE could inform prognosis in ARDS remains unknown. In this secondary analysis of the Lung Imaging for Ventilator Setting in ARDS (LIVE) multicenter randomized controlled trial, which evaluated a personalized ventilation strategy tailored to lung morphology, plasma sRAGE was measured upon study entry (baseline) and on days one, two, three, four and six. The association between changes in plasma sRAGE over time and 90-day survival was evaluated. Higher baseline plasma sRAGE (HR per-one log increment, 1.53; 95% CI, 1.16–2.03; *p* = 0.003) and an increase in sRAGE over time (HR for each one-log increment in plasma sRAGE per time unit, 1.01; 95% CI, 1.01–1.02; *p* < 10^−3^) were both associated with increased 90-day mortality. Each 100-unit increase in the plasma sRAGE level per unit of time increased the risk of death at day 90 by 1% in joint modeling. Plasma sRAGE increased over time when a strategy of maximal alveolar recruitment was applied in patients with focal ARDS. Current findings suggest that the rate of change in plasma sRAGE over time is associated with 90-day survival and could be helpful as a surrogate outcome in ARDS.

## 1. Introduction

Acute respiratory distress syndrome (ARDS), as clinically defined [1], includes patients with a wide range of underlying biologic processes. The identification of physiologic, clinical, and biologic characteristics that define ARDS subgroups or endotypes may add value to determining prognosis and predicting response to treatments [2,3]. Current evidence supports the role of biomarkers in better understanding pathophysiology of lung injury and repair, improving ARDS diagnosis and risk stratification, informing heterogeneity within ARDS, and the testing of novel therapeutic targets [4,5]. However, the potential value of biomarkers as surrogate outcomes for monitoring responses to ventilator settings in patients with ARDS remains under investigated [6].

Elevated plasma levels of soluble receptor for advanced glycation end-products (sRAGE) may reflect the severity of lung epithelial injury [7,8] and are associated with mortality in acute respiratory distress syndrome (ARDS) when measured at baseline [9]. This hypothesis has been supported by studies in ex vivo human lungs [10] and in patients with ARDS [8]. In a recent multicenter observational study, plasma sRAGE was higher in nonfocal ARDS than in focal ARDS [11], possibly suggesting that nonfocal ARDS could be associated with a more severely injured lung alveolar epithelium [12]. Although single measurements of plasma sRAGE could improve our ability to forecast prognosis and identify lung imaging phenotypes within ARDS [9,11], whether changes in plasma sRAGE, which may indicate ongoing alveolar epithelial injury, can inform prognosis and be influenced by some interventions, such as mechanical ventilation, remains unknown.

This secondary analysis of a recent randomized controlled trial [13] was designed to evaluate whether the evolution of lung epithelial injury, as measured by changes in plasma sRAGE over the first days from ARDS onset, could inform prognosis in patients with ARDS. We hypothesized that an increase in plasma sRAGE over the first few days of ARDS would be associated with decreased 90-day survival. In addition, we aimed to investigate the effects on plasma sRAGE kinetics of a personalized strategy of mechanical ventilation tailored to lung morphology, compared to a more conventional low tidal volume and low positive end-expiratory pressure (PEEP) strategy, asking whether these effects would differ between focal and nonfocal ARDS.

Some of the results of this study have been previously reported in the form of an abstract or oral communication during the International ARDS Conference (2019) and the American Thoracic Society International Conference (2020) [14].

## 2. Materials and Methods

Additional details are provided in the Supplementary Materials.

### 2.1. Study Patients

Data for this analysis were obtained prospectively from 235 patients enrolled in the Lung Imaging for Ventilator Setting in ARDS (LIVE) trial within the first 12 h of moderate–severe ARDS [13]. The primary outcome of the LIVE trial was 90-day survival; in the intention-to-treat analysis, a personalized ventilation strategy tailored to lung morphology did not improve survival. Post-hoc reclassification revealed misclassification of lung morphology in 21% of patients during randomization and the subgroup analysis suggested that a personalized ventilator strategy that mismatched the pre-specified trial intervention (i.e., the application of the personalized ventilation strategy initially planned for focal ARDS to patients with nonfocal ARDS, and vice versa) could increase mortality. Ethics (CPP-Sud-Est-VI) and Medicine (ANSM) committees approved the protocol and all patients, or their surrogates, provided written informed consent.

### 2.2. Assay Procedures

Plasma sRAGE was measured in duplicate using enzyme-linked immunosorbent assay kits (RAGE Quantikine, R&D Systems, Minneapolis, MN, USA) at baseline (after randomization and before initiation of study interventions) and on days one, two, three, four, and six when samples were available. The personnel responsible for performing sRAGE assays had no knowledge of the clinical data or of the randomization group.

Plasma sRAGE was not measured in all patients enrolled in LIVE due to limited plasma availability. Only patients with available sRAGE measurements at baseline (day zero) were included in this secondary analysis.

### 2.3. Study Outcomes

The primary outcome was 90-day survival. Secondary outcomes included 28-day survival, ventilator-free days at day 28 (VFD28), and clinical indices of overall severity (sequential organ failure assessment score (SOFA), the need for vasopressor use or continuous renal replacement therapy), the need for rescue ARDS therapies, measures of pulmonary physiologic impairment (PaO_2_/FiO_2_, compliance of the respiratory system), and changes in plasma sRAGE during the first six days after randomization.

### 2.4. Statistical Analysis

Two models were applied to evaluate the association between longitudinal measures of plasma sRAGE and survival. First, sRAGE was considered both at baseline and as a time-varying covariate in a time-varying covariate Cox (TVC) model. Second, joint modeling of longitudinal measures of sRAGE and survival was performed. In both models, a multivariable adjustment was performed on any potential risk confounders as was done in the parent LIVE trial [13,15,16]. Two sensitivity analyses were conducted on both survival models to account for missing data using the last observation carried forward (LOCF) and, for potential immortal bias, limiting patients to those who survived within 3 days after randomization. Associations between changes in sRAGE over time and other continuous or binary outcomes were tested using a multilevel mixed effects generalized linear model and the association between plasma sRAGE and the SOFA score at baseline was assessed using Spearman’s correlation coefficient. Random effects models were used to study the longitudinal evolution of plasma sRAGE [17]. All analyses were performed using Stata (version 15, StataCorp, College Station, TX, USA), and a *p* value of <0.05 (two-sided) was considered statistically significant.

## 3. Results

### 3.1. Study Cohort

**Figure 1:**
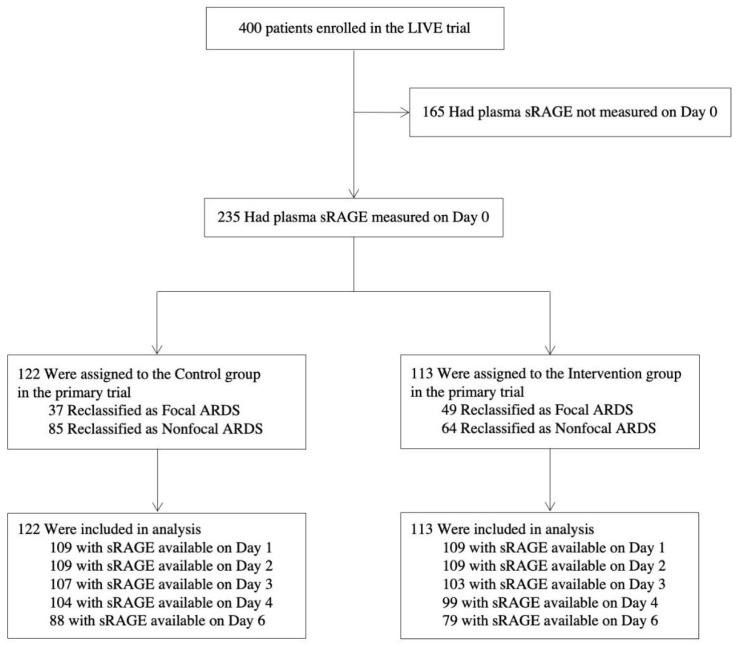
Flow Chart of the Ancillary Study. ARDS: acute respiratory distress syndrome. LIVE: Lung Imaging for Ventilator setting in ARDS (intervention group). sRAGE: soluble receptor for advanced glycation end-products.

Baseline plasma samples were available for sRAGE measurements in 235 of the 400 original clinical trial subjects (Figure 1). The baseline characteristics and clinical outcomes of patients with available sRAGE measurements randomized to the intervention group or to the control group are reported in Table S1 (Supplementary Materials). The clinical characteristics and clinical outcomes of subjects who had plasma samples available were similar to those who did not (Supplementary Materials Table S2), except that patients with plasma sRAGE measured showed lower incidences of pneumonia and pulmonary causes of ARDS, lower incidence of the pre-existing need for chronic dialysis and of no pre-existing medical condition, higher mean pulmonary compliance and lower mean heart rate, PaCO_2_, inspiratory plateau pressure, and driving pressure. 

### 3.2. Changes in Plasma sRAGE and 90-Day Survival

The baseline characteristics of 90-day survivors versus non-survivors are reported in Table 1. In the TVC model, both higher baseline plasma sRAGE (HR per one-log increment, 1.53; 95% CI, 1.16 to 2.03; *p* = 0.003) and the change in plasma sRAGE over time (HR for each one-log increase per unit of time (such as from baseline to day 1, from day 1 to day 2, and so on), 1.01; 95% CI, 1.01 to 1.02; *p* < 10^−3^) were associated with death by day 90 (*n* = 1221 repeated measures from 235 patients), even after multivariable adjustment (Table 2). In the sensitivity analyses of the TVC model, similar effects were obtained when LOCF was used to impute missing data (*n* = 1324 repeated measures from 235 patients; HR per one-log increment in baseline sRAGE, 1.52, 95% CI, 1.16 to 2.00, *p* = 0.002; HR for each one-log increase in sRAGE per unit of time, 1.01; 95% CI, 1.01 to 1.02; *p* < 10^−3^) or when the analysis was restricted to sRAGE values from days 0, 1, 2, and 3 in patients who survived to day 3 (*n* = 807 repeated measures in 213 patients; HR per one-log increment in baseline sRAGE, 1.52, 95% CI, 1.01 to 2.28, *p* = 0.045; HR for each one-log increase in sRAGE per unit of time, 1.01; 95% CI, 1.01 to 1.02; *p* = 0.006), even after multivariable adjustments (Supplementary Materials Tables S3 and S4).

**Figure 2:**
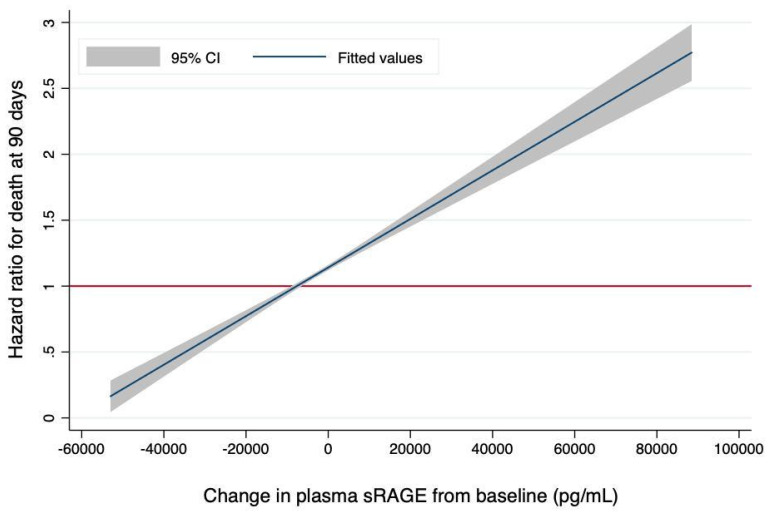
The Risk of Death at 90 Days is Associated with the Magnitude of Change in Plasma sRAGE (in pg·mL^−1^) from Baseline. The hazard ratio for death was computed using joint modeling of longitudinal measurements of sRAGE and 90-day survival and reported on the y-axis for each one-point change in plasma sRAGE (pg·mL^−1^) per unit of time (hazard ratio per 100-unit increase in time-dependent sRAGE, 1.01; 95% confidence interval [CI], 1.01–1.01). The gray shaded areas represent 95% CIs for estimated hazard ratios. sRAGE: soluble receptor for advanced glycation end-products.

Joint modeling confirmed the association between changes in plasma sRAGE and the risk of death at day 90 (HR for each one-log increase in time-dependent plasma sRAGE, 1.55, 95% CI, 1.19 to 2.03, *p* = 0.001), even after adjustment for the same covariates used previously (HR for each one-log increase in time-dependent plasma sRAGE, 2.12, 95% CI, 1.55 to 2.92, *p* < 10^−3^). This corresponded to a 1% increase in the risk of death at day 90 for each 100-unit increase in time-dependent, non-log-transformed plasma sRAGE (Figure 2). The longitudinal trajectory of joint longitudinal measurements of plasma sRAGE and 90-day survival data is illustrated by Figure S1 (Supplementary Materials). Sensitivity analyses of the joint model showed similar trends after LOCF imputation (HR for each one-log increase in time-dependent plasma sRAGE in univariate and multivariable analyses, 7.03, 95% CI, 1.07 to 46.06, *p* = 0.04 and 51.42, 95% CI, 5.99 to 441.42, *p* < 10^−3^, respectively) or when only considering sRAGE values from days 0, 1, 2, and 3 in survivors to day 3 (HR for each one-log increase in time-dependent plasma sRAGE in univariate and multivariable analyses, 1.65, 95% CI, 1.21 to 2.25, *p* = 0.002 and 2.59, 95% CI, 1.70 to 3.90, *p* < 10^−3^, respectively).

**Figure 3:**
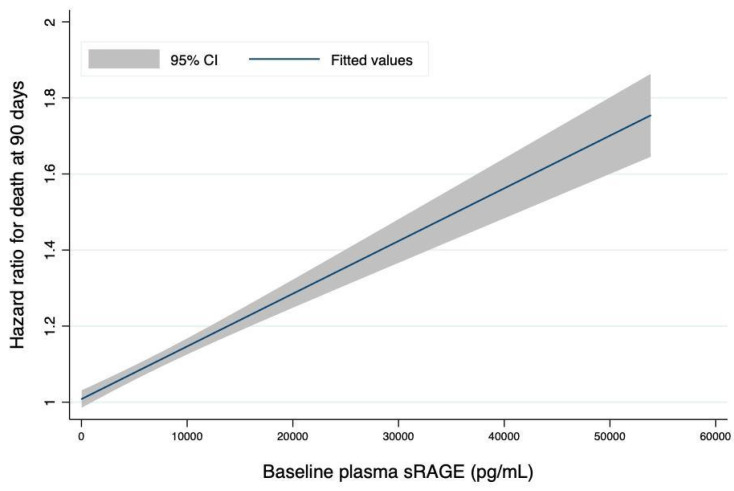
The Risk of Death at 90 Days is Associated with the Magnitude of Change in Plasma sRAGE from Baseline is Increased with Higher Values of Plasma sRAGE at Baseline. The hazard ratio for death was computed using joint modeling of longitudinal measurements of sRAGE and 90-day survival and reported on the y-axis for each one-point increase in plasma sRAGE (pg·mL^−1^) per unit of time. The x-axis represents plasma sRAGE as measured at baseline and expressed in (pg·mL^−1^). The gray shaded areas represent 95% CIs for estimated hazard ratios. sRAGE: soluble receptor for advanced glycation end-products.

The association between the rate of change in sRAGE over time and 90-day survival was increased with higher values of baseline sRAGE (Figure 3).

### 3.3. Changes in Plasma sRAGE and Other Clinical Outcomes

There was a significant association between the rate of change in plasma sRAGE over time and the risk of death at day 28 in the joint model (HR for each one-log increase in time-dependent plasma sRAGE, 1.34, 95% CI, 1.20 to 1.51, *p* < 10^−3^), but not in the TVC model (HR per one-log increment in baseline sRAGE, 1.73; 95% CI, 1.26 to 2.36; *p* = 0.001 and HR for each one-log increase per unit of time, 1.02; 95% CI, 1.00 to 1.05; *p* = 0.1). Using multilevel mixed effects generalized linear models, the rate of increase in sRAGE over time had a poor correlation with fewer VFD28 (regression coefficient, −0.18; 95% CI, −0.25 to −0.11; *p* < 10^−3^), lower daily SOFA scores up to day 6 (regression coefficient, 0.07; 95% CI, 0.04 to 0.10; *p* < 10^−3^), and a weak correlation with the need, as recorded up to day 6, for rescue ARDS therapies (regression coefficient, 0.25; 95% CI, 0.05 to 0.45; *p* = 0.013), vasopressor use (regression coefficient, 0.41; 95% CI, 0.21 to 0.61; *p* < 10^−3^), and continuous renal replacement therapy (regression coefficient, 0.44; 95% CI, 0.18 to 0.71; *p* < 10^−3^).

### 3.4. Plasma sRAGE and Indices of Severity and Lung Injury

Higher baseline plasma levels of sRAGE were associated with more severe ARDS, as reflected by measures of pulmonary physiologic impairment, including lower PaO_2_/FiO_2_ and lower compliance of the respiratory system, and had a poor correlation with higher SOFA scores at baseline (Spearman’s rho = 0.11, *p* < 10^−3^) (Supplementary Materials Figure S2). When measured during the first 6 days after randomization, plasma sRAGE levels had a poor correlation with PaO_2_/FiO_2_ (regression coefficient, −0.02; 95% CI, −0.03 to −0.02; *p* <10^−3^) and with the compliance of the respiratory system (regression coefficient, −0.03; 95% CI, −0.04 to −0.02; *p* < 10^−3^) in multilevel mixed effects generalized linear models (Supplementary Materials Figure S3).

### 3.5. Effects of Ventilator Settings on Plasma sRAGE

Lung morphology was correctly classified by the investigators in the parent clinical trial in 178 (76%) patients included in this analysis. After reclassification of the otherwise 57 (24%) misclassified patients, focal ARDS was identified in 86 (37%) patients and nonfocal ARDS in 149 (63%) patients. The clinical characteristics and clinical outcomes of patients with focal ARDS were similar to those with nonfocal ARDS (Supplementary Materials Table S5), except those with focal ARDs had a lower rate of antibiotic usage at baseline and higher body mass index values. Patients with focal ARDS (as confirmed after reclassification in LIVE) had lower baseline plasma sRAGE (median [IQR], 2346 [(1133–3218) pg·mL^−1^) than those with nonfocal ARDS (3577 (2113–8480) pg·mL^−1^) (*p* = 0.0001) (standardized mean difference [95% CI], 0.54 (0.81–0.27)).

**Figure 4:**
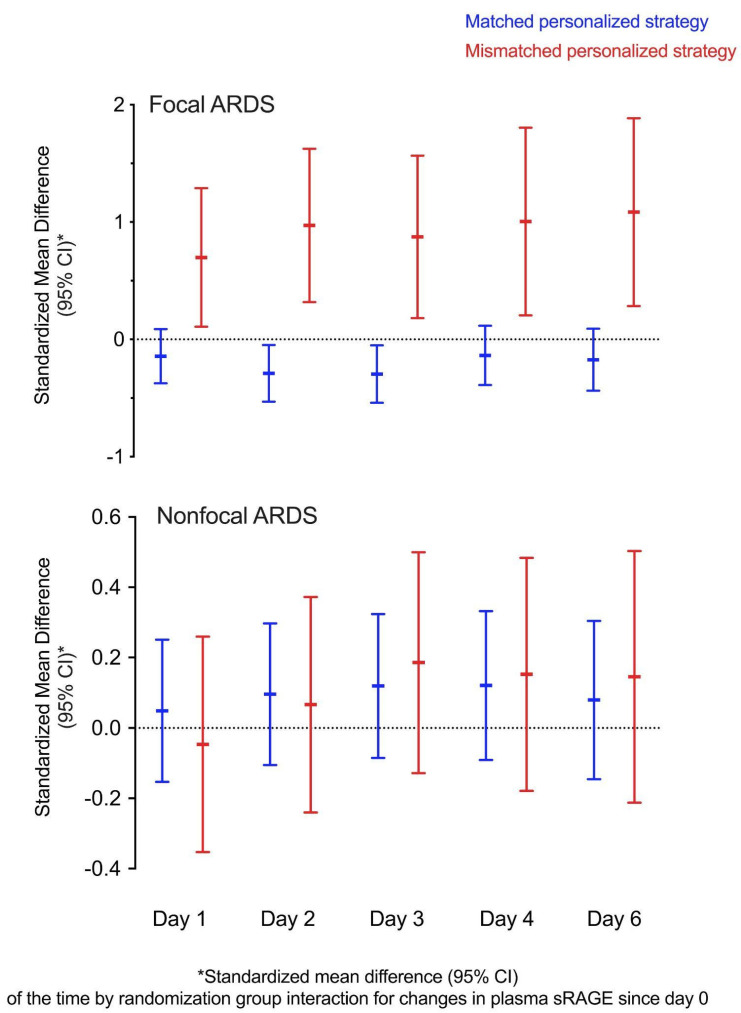
Standardized Mean Differences (with 95% Confidence Intervals [CI]) as a Measure of the Effect Size of the Time by Randomization Group Interactions for Changes in Plasma sRAGE (in pg·mL^−1^) since Day 0 in Patients with Focal (top panel) and Nonfocal (bottom panel) ARDS, Whether the Personalized Ventilator Strategy Matched the Prespecified LIVE Trial Intervention (in blue) or Not (in red). sRAGE: soluble receptor for advanced glycation end-products. ARDS: acute respiratory distress syndrome. LIVE: Lung Imaging for Ventilator Setting in ARDS.

We analyzed the effects of the ventilation strategy on the changes in plasma sRAGE. There was a significant time by group interaction leading to a decrease in plasma sRAGE on days two and three (*p* = 0.02 for both timepoints) in patients with focal ARDS when the personalized strategy matched the intervention tailored to lung morphology as initially planned in the LIVE trial. In contrast, a significant time by group interaction resulted in increased plasma sRAGE in patients with focal ARDS on days one, two, three, four, and six (*p* = 0.02, 0.004, 0.01, 0.01, and 0.001, respectively) when lung morphology was incorrectly classified and the mismatched personalized ventilation strategy was applied (Supplementary Materials Table S6). In patients with nonfocal ARDS, there was no significant time by group effect on plasma sRAGE kinetics, regardless of whether the personalized ventilation strategy matched lung morphology or not. Effect sizes for time by group interactions on changes in plasma sRAGE are reported as standardized mean differences in Figure 4.

## 4. Discussion

The novel finding of this study is that the rate of change in plasma sRAGE was associated with survival; specifically, increases in sRAGE over the first days following ARDS onset were associated with worse clinical outcomes. Schematically, in this study, each 100-unit increase in the plasma sRAGE level (as expressed in pg·mL^−1^) per unit of time increased the risk of death at day 90 by 1%. In addition, the 90-day risk of death associated with increases in sRAGE over time was greater when the baseline sRAGE level was higher [9,18].

The finding of a significant association between the rate of change in plasma sRAGE over time and clinical outcomes was rather consistent across multiple analyses. These analyses included joint (simultaneous) modeling of longitudinal and time-to-event data, which is considered to be the best method for evaluating the relationship between a biomarker trajectory and clinical outcomes such as survival because it accounts for immortal time bias and, therefore, allows for informative dropouts such as death. Notably, the rate of change in plasma sRAGE was associated with VFD28 and other indices of clinical severity, such as the SOFA score and the need for rescue ARDS therapies, vasopressor use, and continuous renal replacement therapy over the first days following ARDS onset. Previous evidence indicates that baseline plasma sRAGE levels are associated with measures of lung injury severity and impaired alveolar fluid clearance [8,11,18,19,20]. However, the current analysis does not support relevant associations between plasma sRAGE and some indices of lung injury severity, suggesting that changes in sRAGE over time (reflecting changes in the degree of lung epithelial injury) might provide novel prognostic information compared to that related to the changes in conventional mechanisms of oxygenation or respiratory system compliance [9,21].

In this analysis, focal ARDS were characterized by a lower baseline plasma sRAGE than nonfocal ARDS, and a higher baseline plasma sRAGE was associated with the severity of ARDS, supporting previous evidence [11,18,20,22]. We also found that, compared to the low-PEEP, low-Vt control strategy, the so-called personalized ventilation strategy used in LIVE had distinct effects on the temporal course of plasma sRAGE in focal and nonfocal ARDS when there were mismatches in the pre-specified trial interventions resulting from the misclassification of lung morphology by local investigators during randomization. In focal ARDS, the use of the mismatched personalized strategy combining a Vt of 8 mL·kg^−1^ predicted body weight (PBW), lower PEEP, and early prone position (PP) [13] was associated with decreased plasma sRAGE levels by days 2–3, whereas the strategy of maximal alveolar recruitment combining a Vt of 6 mL·kg^−1^ PBW, higher PEEP, and repeated recruitment maneuvers (RM) was associated with increased plasma sRAGE levels from day 1 to day 6, suggesting increased injury to the lung alveolar epithelium possibly caused by hyperinflation and lung mechanical stress [23,24]. In contrast, in nonfocal ARDS, the mismatched personalized strategy was not associated with increases in plasma sRAGE over time compared to the control strategy, suggesting that the use of lower Vt and PEEP and PP may not cause or amplify lung epithelial injury per se in nonfocal ARDS compared to a strategy combining higher PEEP and RM. This is consistent with previous reports suggesting that a lower Vt (6 mL·kg^−1^ PBW) ventilation may amplify the decrease in sRAGE over time in unselected ARDS compared to a higher Vt (12 mL·kg^−1^ PBW) ventilation [18] and that applying an RM is associated with an early and transient decrease in plasma sRAGE in nonfocal ARDS [6]. Unfortunately, the ventilation strategies from LIVE were based on multimodal interventions (Vt, PEEP, PP, RM), which made it impossible to assess the precise effects of each intervention on sRAGE kinetics in our study. However, combined with the fact that the rate of change in sRAGE is associated with prognosis, these current hypothesis-generating findings suggest that longitudinal measurements of plasma sRAGE should be further explored as potential tools to assess treatment response in future enriched ARDS trials [2].

This study has limitations. First, sRAGE was measured only at pre-specified timepoints (days 0, 1, 2, 3, 4, and 6), and rapid, short-term changes in plasma sRAGE could have been missed [6]. However, the logistics of conducting a study that would integrate more frequent sRAGE measurements remain highly challenging. Second, we measured plasma but not alveolar sRAGE. Previous reports have shown that a bronchoalveolar lavage fluid analysis was more sensitive for detecting local damage to the lung epithelium because the alveolar sRAGE level is higher than the plasma sRAGE level when epithelial injury causes the shedding of sRAGE into the alveolar space [7]. However, plasma sRAGE has been recognized as a valuable marker of lung epithelial injury and is more readily sampled at serial timepoints [7,8]. Third, sRAGE was measured in subjects with available plasma samples only, not in all patients enrolled in the primary LIVE trial. Although there were only a few differences between patients who had plasma sRAGE measured and those who did not, we cannot exclude the possibility that unmeasured factors may have contributed to some degree of inclusion bias. However, a previous study found no association between plasma levels of sRAGE and clinical and biological indices that are usually recorded upon ICU admission. Finally, measurements were limited to sRAGE in this analysis, and it remains unknown as to how other previously reported ARDS biomarkers, such as markers of inflammation [25,26,27,28,29] would compare to lung imaging phenotypes. Our findings also further support the recognition of focal and nonfocal ARDS as distinct ARDS phenotypes [3,29]. These findings could, therefore, be useful in future trials designed to test prospectively the logistics of incorporating clinical, imaging, and biological measurements in order to facilitate population enrichment in future trials [2,29,30], although this would ideally require development of a point-of-care test to measure sRAGE. Such advancements would facilitate the development of more personalized approaches to managing patients with ARDS.

In this secondary analysis of 235 patients with ARDS, the rate of change in plasma sRAGE over time was associated with 90-day survival. These hypothesis-generating findings should foster future research to determine whether plasma sRAGE can be used as a treatable biological trait or surrogate outcome in ARDS.

## Figures and Tables

**Table 1:** Baseline Characteristics and Clinical Outcomes of Survivor and Non-survivor Patients with ARDS at Day 90.

Characteristic	Survivors	Non-Survivors	*p* Value
	(*n* = 165)	(*n* = 70)	
*Demographics*			
Male sex, n (%)	122 (74)	55 (79)	0.5
Age, years	60 ± 15	67 ± 13	10^−4^
BMI, kg.m^−2^	26 ± 5	26 ± 5	0.7
*Coexisting Conditions, n (%)*			
COPD	14 (8)	7 (10)	0.8
Hematologic neoplasm	5 (3)	3 (4)	0.7
Chronic dialysis	0 (0)	0 (0)	1
Other	122 (74)	59 (84)	0.09
None	39 (23)	7 (10)	0.01
*Indication for ICU Admission, n (%)*			0.04
Septic shock	27 (16)	12 (17)	
Hemorrhagic shock	3 (2)	4 (6)	
Coma	5 (3)	0 (0)	
Trauma	5 (3)	0 (0)	
Acute respiratory failure	49 (30)	15 (21)	
Elective surgery	19 (12)	3 (4)	
Emergent surgery	8 (5)	3 (4)	
Other	49 (30)	33 (47)	
*Cause of ARDS, n (%)*			0.5
Pulmonary	115 (70)	46 (66)	
Extrapulmonary	50 (30)	24 (34)	
*Baseline Respiratory Variables*			
PEEP, cmH_2_O	10 ± 3	11 ± 4	0.1
Tidal volume, mL·kg^−1^PBW	6.7 ± 1.2	6.5 ± 1.1	0.3
Respiratory rate, per min	24 ± 5	25 ± 5	0.2
Pplat, cmH_2_O	23 ± 5	24 ± 5	0.3
Static pulmonary compliance, mL·cmH_2_O^−1^	37 ± 16	37 ± 17	0.8
Driving pressure, cmH_2_O	13 ± 5	13 ± 5	0.7
PaO_2_, mmHg	87 ± 29	83 ± 28	0.4
PaO_2_/FiO_2_, mmHg	120 ± 41	111 ± 40	0.2
PaO_2_/FiO_2_ <100 mmHg, *n* (%)	62 (38)	29 (41)	0.6
PaCO_2_ mmHg	43 ± 9	47 ± 12	0.01
FiO_2_, %	75 ± 20	78 ± 21	0.3
Arterial pH	7.34 ± 0.10	7.28 ± 0.12	0.0006
Serum bicarbonate, mmol.L^−1^	22 ± 5	19 ± 5	0.02
*Baseline Hemodynamic Status*			
Mean arterial blood pressure, mmHg	79.0 ± 13.4	76.7 ± 15.2	0.06
Heart rate, per min	96 ± 24	99 ± 20	0.3
Serum lactate, mmol·L^−1^	2.3 ± 3.1	3.4 ± 2.6	0.0001
Need for norepinephrine, *n* (%)	98 (59)	54 (77)	0.01
*Baseline Renal Status*			
Serum creatinine, μmol·L^−1^	123 ± 88	159 ± 93	0.0004
Need for renal replacement therapy, *n* (%)	5 (3)	9 (13)	0.003
*Baseline Septic Status*			
Under antibiotic therapy, *n* (%)	71 (83)	138 (93)	0.02
Abdominal sepsis, *n* (%)	17 (20)	23 (15)	0.4
Urinary tract infection, *n* (%)	0 (0)	5 (3)	0.1
Pneumonia, *n* (%)	43 (50)	98 (66)	0.02
Septicemia, *n* (%)	2 (2)	1 (1)	0.6
Soft tissue infection	1 (1)	0 (0)	0.5
Other infection	21 (13)	7 (10)	0.6
*Corticosteroid therapy, n (%)*	34 (21)	20 (29)	0.2
*Serum bilirubin, μmol·L^−1^*	22 ± 35	36 ± 54	0.009
*Baseline Severity of Illness*			
SAPS II	48 ± 16	59 ± 17	0.0001
SOFA	9 ± 3	11 ± 4	0.0001
McCabe classification, *n* (%)			
Category 1: Nonfatal disease	116 (72)	35 (52)	0.02
Category 2: Ultimately fatal disease	42 (26)	29 (43)	
Category 3: Rapidly fatal disease	4 (2)	3 (4)	
*Plasma sRAGE, pg·mL^−1^ (median (interquartile))*			
Baseline (day 0)	3021 (1597–4663)	3245 (1892–5810)	0.2
Day 1	1962 (1064–3413)	2357 (1350–4430)	0.06
Day 2	1385 (827–2398)	1660 (920–3409)	0.1
Day 3	1303 (689–2074)	1427 (821–2275)	0.3
Day 4	1196 (674–2171)	1343 (524–2064)	0.9
Day 6	1139 (581–1757)	1170 (497–2355)	0.7
*Lung morphology, n (%)*			0.4
Focal ARDS	63 (38)	23 (33)	
Nonfocal ARDS	102 (62)	47 (67)	
*Randomization group, n (%)*			0.9
Control	85 (52)	37 (53)	
Intervention	80 (48)	33 (47)	

Data are presented as mean ± standard deviation (SD) unless otherwise indicated. P-values were calculated for comparisons between patients with survivors and non-survivors. Percentages may not exactly total 100% because of rounding. The body mass index (BMI) is the weight in kilograms divided by the square of the height in meters. COPD: chronic obstructive pulmonary disease. ICU: intensive care unit. ARDS: acute respiratory distress syndrome. PEEP: positive end-expiratory pressure. Pplat: inspiratory plateau pressure. PaO_2_: partial pressure of arterial oxygen. FiO_2_: fraction of inspired oxygen. SAPS II: simplified acute physiology score II. SOFA: Sequential Organ Failure Assessment score. sRAGE: soluble receptor for advanced glycation end-products.

**Table 2:** Multivariable Marginal Cox Survival Analyses of Death at Day 90, Considering Plasma sRAGE both at Baseline and as a Time-varying Covariate.

	Hazard Ratio (95% CI)	*p*
Baseline plasma sRAGE *	1.53 (1.16–2.03)	0.003
Increase in plasma sRAGE **	1.01 (1.01–1.02)	<10^−3^
Baseline plasma sRAGE *	1.47 (1.17–1.84)	0.001
Increase in plasma sRAGE **	1.01 (1.01–1.02)	0.006
Age–yr	1.01 (0.99–1.05)	0.05
SAPS II	1.03 (1.01–1.05)	0.01
McCabe category 2	1.58 (0.85–2.95)	0.15
McCabe category 3	0.96 (0.27–3.39)	0.9
History of hematologic cancer	0.68 (0.18–2.50)	0.6
History of solid cancer	5.01 (2.24–11.20)	<10^−3^
Shock at baseline	1.34 (0.64–2.77)	0.4
Need for continuous renal replacement therapy at baseline	1.53 (0.69–3.40)	0.3
Corticosteroid therapy at baseline	0.87 (0.51–1.84)	0.9
Randomization to the personalized ventilation group	1.03 (0.57–1.86)	0.9
Focal lung morphology (after post-hoc reclassification)	0.87 (0.45–1.66)	0.7
Correct classification of lung morphology at baseline	0.30 (0.16–0.59)	<10^−3^

* Hazard Ratio is expressed for each one-log increment in baseline plasma sRAGE. ** Hazard Ratio is expressed for each one-log increase in plasma sRAGE per unit of time. *n* = 1174 repeated sRAGE measures from 235 patients available for complete-case multivariable analysis. SAPS II: simplified acute physiology score II. sRAGE: soluble receptor for advanced glycation end-products.

## Data Availability

Data collected for the study, including individual participant data, a data dictionary defining each field in the set, and the study protocol are stored at the Direction de la Recherche Clinique, CHU Clermont-Ferrand, Clermont-Ferrand, France. De-identified data will be made available upon reasonable request after publication of the manuscript.

## Supplementary Materials

The following are available online at https://www.mdpi.com/article/10.3390/jcm10102076/s1, Figure S1: Longitudinal Trajectory Plot of Joint Longitudinal Measurements of Plasma sRAGE and 90-Day Survival Data, in Patients who were Censored (*left panel*) and in Those who Experienced the Event of Interest (Death) (*right panel*), Figure S2: Higher Plasma sRAGE (in pg·mL^−1^) is Associated with More Severe Disturbances in Pulmonary Physiology at Baseline: (A) PaO_2_/FiO_2_ (in mmHg) (n = 235), (B) Compliance of the respiratory system (in mL·cmH_2_O^−1^) (n = 171). (C) SOFA score vs. Plasma sRAGE (in pg·mL^−1^) when Measured During at Baseline, Figure S3: (A) PaO_2_/FiO_2_ (mmHg) vs. Plasma sRAGE (pg·mL^−1^, log-transformed) when Measured During the First 6 Days after Randomization (regression coefficient, −0.02; 95% CI, −0.03 to −0.02; *p* < 10^−3^). (B) Compliance of the Respiratory System (mL·cmH_2_O^−1^) vs. Plasma sRAGE (pg·mL^−1^, log-transformed) when Measured During the First 6 Days after Randomization (regression coefficient, −0.03; 95% CI, −0.04 to −0.02; *p* < 10^−3^), Table S1: Baseline Characteristics and Clinical Outcomes of Patients with ARDS Randomized to the Intervention (LIVE) Group or to the Control Group, and Enrolled in the Secondary Analysis, Table S2: Baseline Characteristics and Clinical Outcomes of the LIVE Study Sample, Table S3: Multivariable Marginal Cox Survival Sensitivity Analyses of Death at Day 90, Considering Plasma sRAGE both at Baseline and as a Time-varying Covariate, When Imputing Missing Data Using LOCF was Used to Impute Missing Data, Table S4: Multivariable Marginal Cox Survival Sensitivity Analyses of Death at Day 90, Considering Plasma sRAGE both at Baseline and as a Time-varying Covariate, When Restricting Analyses to Plasma sRAGE values from days 0, 1, 2, and 3 in Patients who Survived to Day 3, Table S5: Baseline Characteristics and Clinical Outcomes of Patients with Focal and Nonfocal ARDS, Table S6: Effects of Time, Group, and Time x Group Interaction on Plasma sRAGE Measured at Study Timepoints, Stratified by Focal vs. Nonfocal Lung Morphology and Matched vs. Mismatched Personalized Ventilation Strategy.

## Abbreviations

ARDS	acute respiratory distress syndrome
BMI	body mass index
COPD	chronic obstructive pulmonary disease
FiO_2_	fraction of inspired oxygen
HR	hazard ratio
ICU	intensive care unit
LIVE	Lung Imaging for Ventilator Setting in ARDS
LOCF	last observation carried forward
PaCO_2_	partial pressure of arterial carbon dioxide
PaO_2_	partial pressure of arterial oxygen
PEEP	positive end-expiratory pressure
Pplat	inspiratory plateau pressure
SAPS II	simplified acute physiology score II
SOFA	sequential organ failure assessment score
sRAGE	soluble receptor for advanced glycation end-products
TVC	time-varying covariate
VFD28	ventilator-free days at day 28
