# Supplementary material for: Changes in Plasma Soluble Receptor for Advanced Glycation End-Products Are Associated with Survival in Patients with Acute Respiratory Distress Syndrome
Source: J Clin Med. 2021 May 12;10(10):2076. doi: 10.3390/jcm10102076 (PMC8150905; doi:10.3390/jcm10102076)
Supplement: Supplementary file 1 [file jcm-10-02076-s001.zip › jcm-1203526-supplementary.pdf]

Journal of
Clinical Medicine

MDPI

SUPPLEMENTARY MATERIALS

MATERIALS AND METHODS

Ethics statements

This secondary analysis was planned a priori and registered prior to inclusion in the LIVE study (clinicaltrials.gov identifier: NCT02149589). The study protocol has been approved by our institutional review board (*Comité de Protection des Personnes Sud Est VI*, approval number AU 1099) and registered by the French *Agence Nationale de Sécurité du Médicament* (approval number 2013-A01756-39). All participants or their surrogates provided written informed consent. There was no deviation from the approved protocol.

Study patients

Clinical data and biological samples for this analysis were obtained prospectively from patients enrolled, within the first 12 h of moderate-to-severe ARDS, in the multicenter investigator-initiated, patient-blinded, randomized, stratified controlled trial of Lung Imaging for Ventilator Setting in ARDS (LIVE) [1]. Patients under mechanical ventilation for more than seven consecutive days during the last 30 days, with history of ARDS in the last month, intracranial hypertension, a body mass index >40 $kg.m^{-2}$, chronic respiratory disease under long-term oxygen therapy, bone marrow transplantation, metastatic cancer, burns, liver cirrhosis with basal Child and Pugh score of C or bronchopleural fistula, pregnant patients, and those already enrolled in another interventional study were ineligible for inclusion in the LIVE trial.

Lung morphology was assessed by local investigators according to the results from CT scan, chest radiograph alone or combined with lung ultrasound (if the patient was considered non-transportable) [2]. A prespecified post-hoc reclassification of lung morphology was performed *a posteriori* by a senior radiologist and two intensivists blinded from the patient history and randomization group [3].

In both groups, low tidal volume (Vt) volume-controlled ventilation was used, and a plateau pressure of ≤30 cmH_2O was targeted. Patients were paralyzed using a continuous intravenous infusion of cisatracurium. In the control group (conventional strategy), PEEP and FiO_2 were set using the low-PEEP ARDSNet table [4] and combined with a Vt of 6 $mL.kg^{-1}$ of predicted body weight (PBW); prone position (PP) was applied as early as possible after inclusion. In the intervention group (personalized strategy), the ventilator was set according to lung morphology (classified as either focal or nonfocal by local investigators during randomization). In patients with focal ARDS, the personalized ventilation strategy combined a Vt of 8 $mL.kg^{-1}$ PBW, along with low PEEP (5-10 cmH_2O) and PP as early as possible. In patients with nonfocal ARDS, the personalized ventilation strategy combined a Vt of 6 $mL.kg^{-1}$ PBW with repeated RM and PEEP set to reach a plateau pressure of 30 cmH_2O [5].

In both groups, as soon as PaO_2/FiO_2 was >200 mmHg for at least 4 h with FiO_2 <60%, or 48 h after inclusion (whichever occurred first), neuromuscular blockers were discontinued, the level of sedation targeted to a Richmond Agitation-Sedation Scale (RASS) of 0/-1, and the ventilator mode switched to pressure-support ventilation (PSV). In case of failure of PSV to maintain adequate gas exchange (PaO_2/FiO_2 >200 mmHg for 4 h with FiO_2 <60%), sedation was increased, neuromuscular blockade was started again (if not already used for more than 48 h since inclusion), and the ventilator set back to volume-controlled ventilation according to the randomization group. Full details of the study design and strategies of mechanical ventilation have been published elsewhere [3].

The primary outcome of the LIVE trial was all-cause 90-day mortality; in this trial, personalization of mechanical ventilation did not decrease mortality in the intention-to-treat survival analysis. Post-hoc reclassification revealed misclassification of lung morphology in 21% of patients during randomization. Subgroup analysis suggested that a personalized ventilator strategy that mismatched the prespecified trial intervention (i.e., the application of the personalized ventilation strategy initially planned for focal ARDS to patients with nonfocal ARDS, and vice versa, due to the initial misclassification of lung morphology) could increase mortality.

Study outcomes

The primary outcome was 90-day survival, as in the primary LIVE trial.
Secondary outcomes included:

- 28-day survival,
- the number of ventilator-free days at day 28 (VFD28), as defined as the number of days from the time of initiating unassisted breathing to day 28 after randomization, assuming survival for at least two consecutive calendar days

after initiating unassisted breathing and continued unassisted breathing to day 28 (if a patient returned to assisted breathing and subsequently achieved unassisted breathing to day 28, VFDs were counted from the end of the last period of assisted breathing to day 28; a period of assisted breathing lasting less than 24 hours, such as for the purpose of a surgical procedure did not count against the VFD calculation; if a patient was receiving assisted breathing at day 27 or died prior to day 28, VFDs were equal to zero),

- indices of overall clinical severity, as recorded daily during the first six days after randomization: sequential organ failure assessment score (SOFA) and the need for vasopressor use and for continuous renal replacement therapy,
- the need for rescue therapies for refractory hypoxemia, as recorded daily during the first six days after randomization and defined as per in the primary LIVE trial: extracorporeal membrane oxygenation, inhaled nitric oxide, neuromuscular blockade for more than 48 h, PP in patients with nonfocal ARDS randomized to the intervention group, and RM in patients randomized to the control group or in those with focal ARDS randomized to the intervention group,
- measures of pulmonary physiologic impairment as recorded daily during the first six days after randomization: PaO_2/FiO_2, compliance of the respiratory system,
- changes in plasma sRAGE over time (as measured on days one, two, three, four, and six after randomization in the LIVE trial).

Statistical analysis

All analyses were performed using Stata (version 15, StataCorp, College Station, TX), and a P-value of <0.05 (two-sided) was considered statistically significant. Because this was a secondary analysis of data collected prospectively from the LIVE randomized clinical trial, no formal sample size was calculated a priori; however, the sample size was considered relevant to assess the prognostic value of changes in plasma sRAGE over time.

Continuous data were expressed as mean and standard deviation (SD) or median and interquartile range [IQR] according to the statistical distribution. The assumption of normality distribution was studied using Shapiro-Wilk's test. To compare continuous characteristics between groups (such as between survivors and non-survivors, focal and nonfocal ARDS or patients with plasma sRAGE available at baseline and those without), Student's t-test or Mann–Whitney test were applied for continuous parameters, whereas categorical data were compared among groups using Chi-square or Fisher's exact tests.

Two distinct models were applied to evaluate the association between longitudinal (plasma sRAGE) and time-to-event data (survival).

First, plasma sRAGE was considered both at baseline and as a time-varying covariate in a time-varying covariate Cox (TVC) model. Censored data were assessed using the Kaplan-Meier approach. The log-rank test and Cox proportional hazards regression were performed for univariate analysis. Marginal Cox model was used for multivariable adjustments on potential risk confounders as was done in the parent LIVE trial [1]; age, severity of illness at baseline as evaluated by the simplified acute physiology score (SAPS) II [6], underlying illness severity as evaluated using the McCabe score [7], history of hematologic cancer or solid cancer, presence of shock at baseline (as defined by the need for vasopressor support), the need for continuous renal replacement therapy, treatment with corticosteroids, randomization to the personalized ventilation strategy in LIVE, lung morphology (focal versus nonfocal), and misclassification of lung morphology during randomization, in addition to center as random-effect. The proportional-hazard hypothesis was verified using Schoenfeld's test and by plotting residuals, and the results were expressed as hazard ratios (HR) and 95% confidence intervals (CI).

Second, because the TVC model does not account for informative missing data or dropouts such as death, joint modeling of longitudinal measures of plasma sRAGE and survival was performed using the *stjm* command in Stata. In this model, it was assumed that the association between longitudinal measurements of sRAGE and survival was underlined by shared random effects, using computationally numerical integration adaptive Gauss–Hermite quadrature to evaluate the likelihood. We also assumed a linear mixed-effects model for the longitudinal submodel, allowing flexibility through the use of fixed or random fractional polynomials of time. Multivariable adjustments were performed using the same covariates than in the TVC model.

Two sensitivity analyses were conducted on both the TVC and the joint models. To account for missing data, last observation carried forward (LOCF) was applied for imputation of missing data. Missing values for plasma sRAGE were filled in with the value that was available from the previous measurement timepoint. In some cases, it implied repeating values from the previous measurement timepoint for multiple subsequent timepoints, which we believe represented a worst-case scenario to evaluate the association between longitudinal sRAGE and survival. Next, to account for potential bias arising from systematic differences in the timing of changes in plasma sRAGE between patients who

died early after randomization and those who did not (immortal time bias), we conducted a sensitivity analysis of the two primary models limiting our analyses to sRAGE levels measured on days 0, 1, 2, and 3 in patients who survived up to day 3 after randomization (landmark analysis).

To test the associations between changes in plasma sRAGE over time and other continuous or binary outcomes (such as VFD28, SOFA, the need for vasopressor use, for continuous renal replacement therapy or for rescue ARDS therapies, PaO_2/FiO_2, and compliance of the respiratory system), we performed a multilevel mixed-effects generalized linear model (*meglm* command in Stata) set to account for the correlation among the repeated measurements taken from the same patient, and considering both within-subject and within-center effects as random-effects. Results were reported as regression coefficients and 95% CI. The association between plasma sRAGE and the SOFA score at baseline was assessed using Spearman's correlation coefficient.

To study the longitudinal evolution of plasma sRAGE, we performed random-effects models using log-transformed values as sRAGE was not normally distributed. Group, time, and their interaction were studied as fixed effects (whereas patient effect was taken into account as random-effect, nested in center effect) and the time by randomization group interactions for changes in plasma sRAGE since day 0 were expressed as standardized mean differences (SMD) with 95% CI. The SMD, also known as Cohen's d, is a measure of the effect size for continuous outcome variables (such as plasma sRAGE in this study) [8].

FIGURES

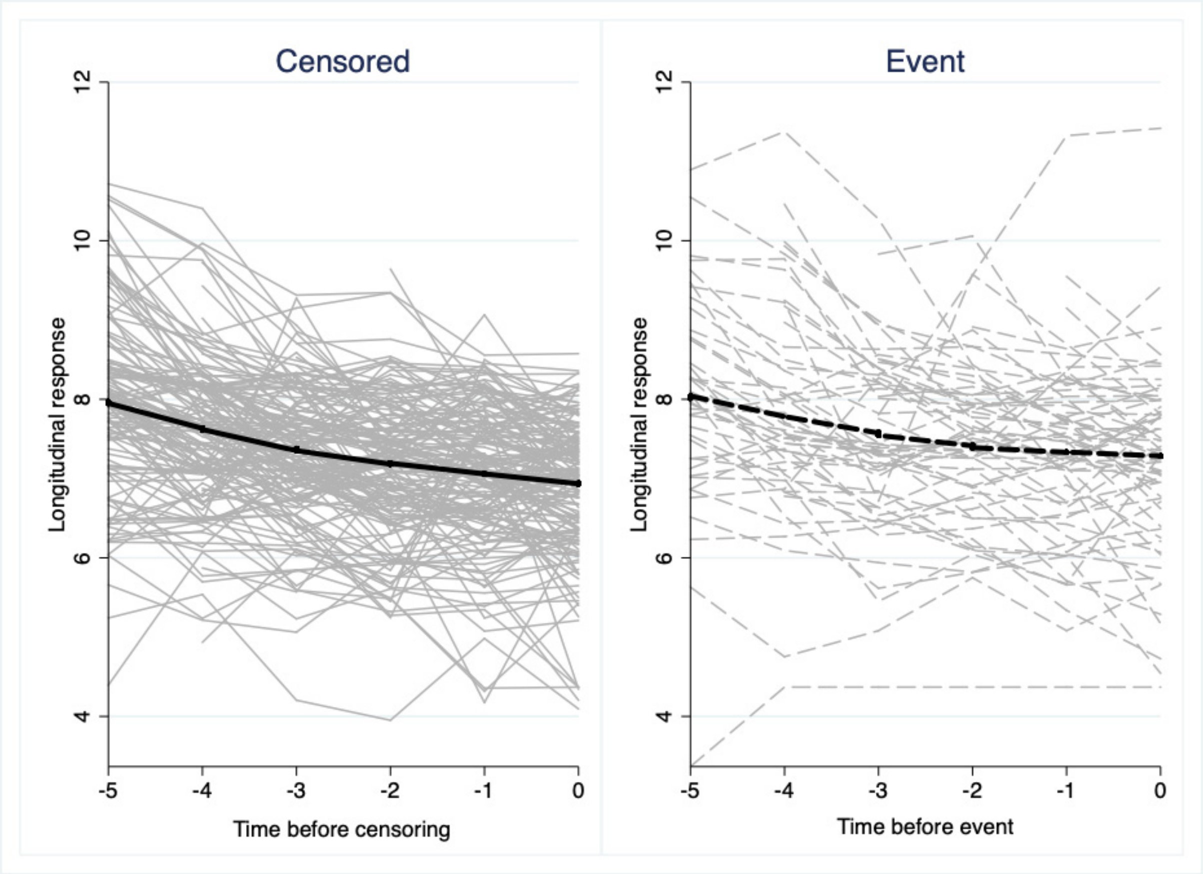

Figure S1. Longitudinal Trajectory Plot of Joint Longitudinal Measurements of Plasma sRAGE and 90-Day Survival Data, in Patients who were Censored *(left panel)* and in Those who Experienced the Event of Interest (Death) *(right panel)*. The timescale is adjusted by taking away each patient's event/censoring time. *sRAGE: soluble receptor for advanced glycation end-products.*

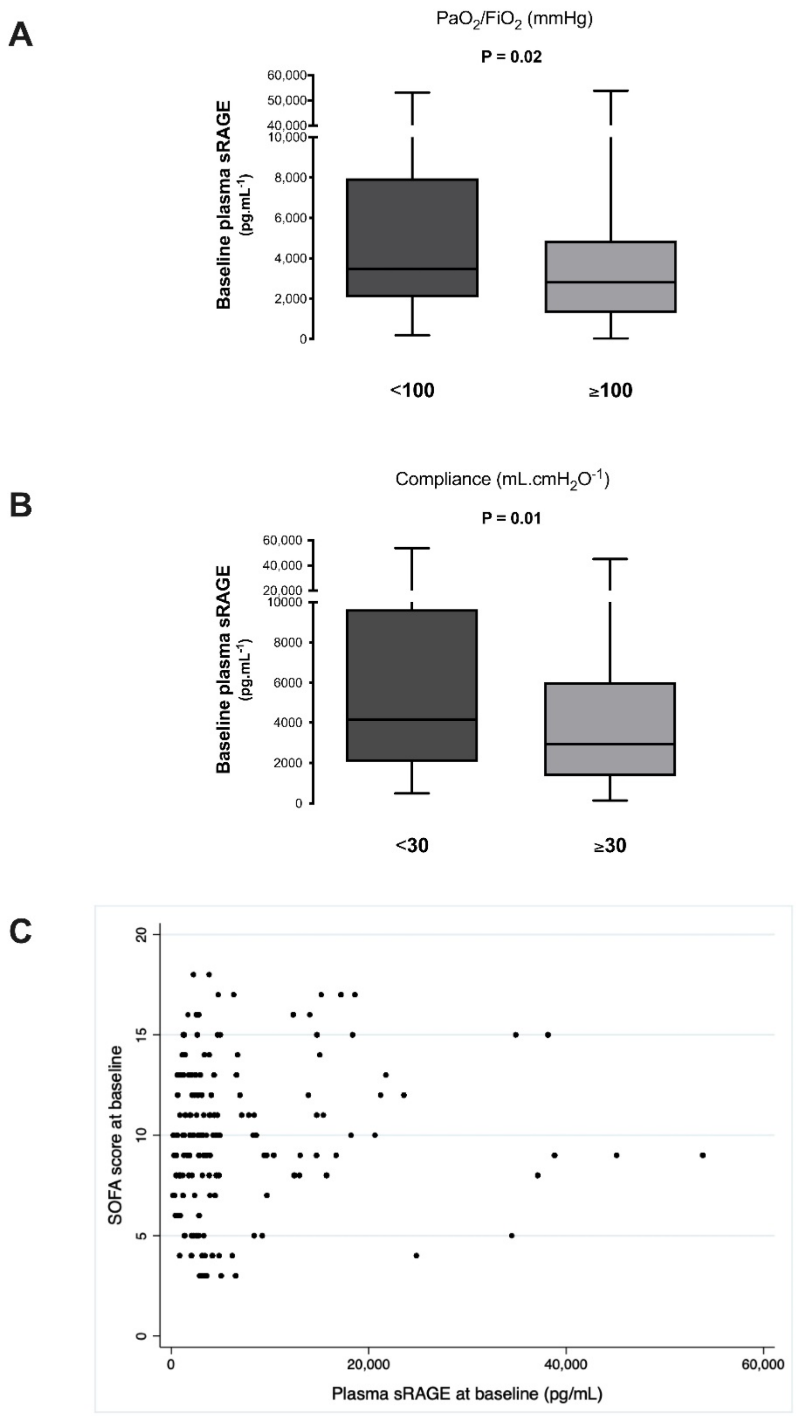

Figure S2. Higher Plasma sRAGE (in pg.mL^{-1}) is Associated with More Severe Disturbances in Pulmonary Physiology at Baseline: A) PaO_2/FiO_2 (in mmHg) (n=235), B) Compliance of the respiratory system (in mL.cmH_2O^{-1}) (n=171). C) SOFA score vs. Plasma sRAGE (in pg.mL^{-1}) when Measured During at Baseline. Boxes show interquartile ranges, error bars indicate 10th to 90th percentiles. *sRAGE: soluble receptor for advanced glycation end-products. SOFA: sequential organ failure assessment score.*

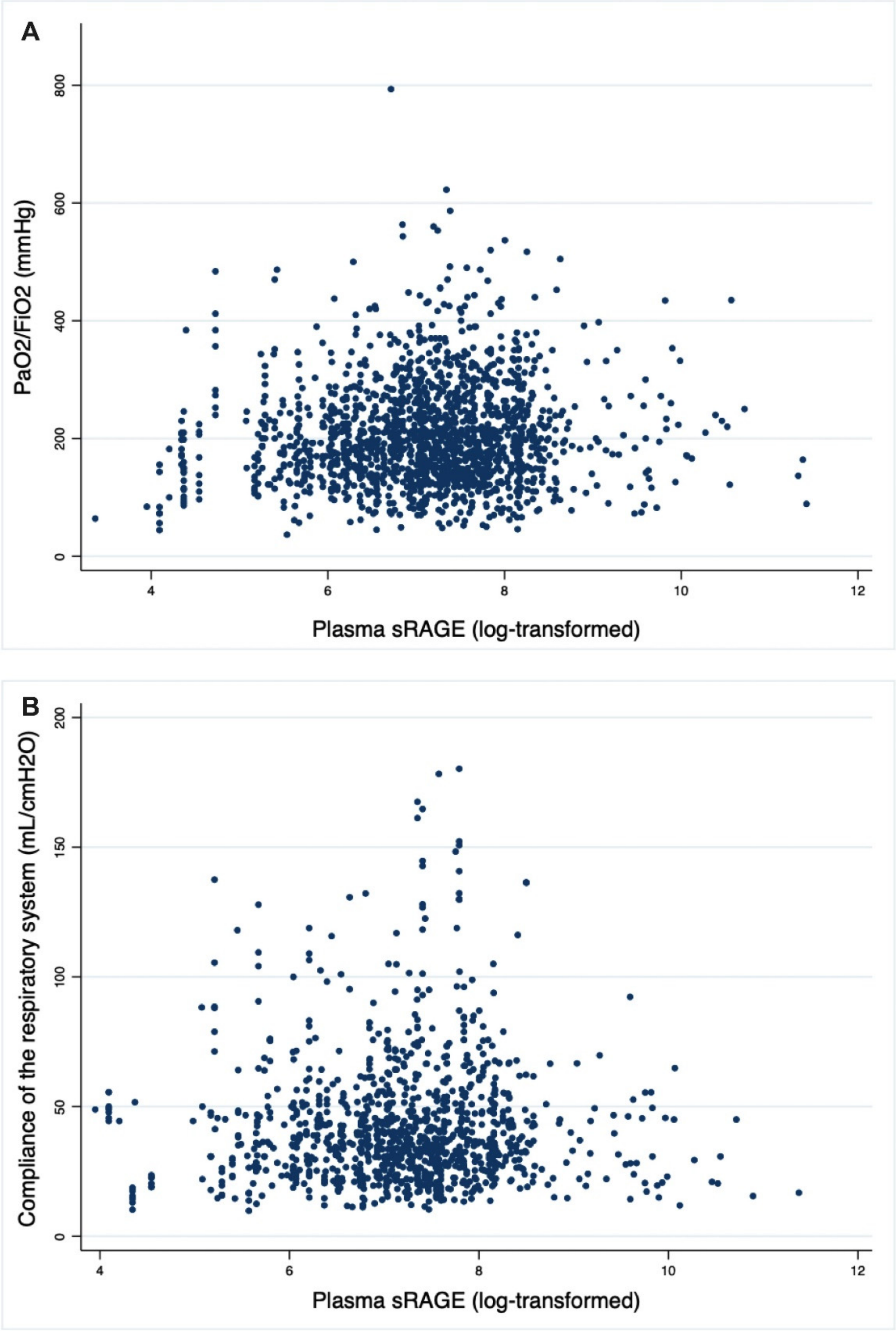

Figure S3. A) PaO_2/FiO_2 (mmHg) vs. Plasma sRAGE ($pg.mL^{-1}$, log-transformed) when Measured During the First 6 Days after Randomization (regression coefficient, -0.02; 95% CI, -0.03 to -0.02; P $<10^{-3}$). B) Compliance of the Respiratory System ($mL.cmH_2O^{-1}$) vs. Plasma sRAGE ($pg.mL^{-1}$, log-transformed) when Measured During the First 6 Days after Randomization (regression coefficient, -0.03; 95% CI, -0.04 to -0.02; P $<10^{-3}$). *sRAGE: soluble receptor for advanced glycation end-products.*

TABLES

Table S1. Baseline Characteristics and Clinical Outcomes of Patients with ARDS Randomized to the Intervention (LIVE) Group or to the Control Group and Enrolled in the Secondary Analysis.

Copyright: © 2021 by the authors. Licensee MDPI, Basel, Switzerland. This article is an open access article distributed under the terms and conditions of the Creative Commons Attribution (CC BY) license (http://creativecommons.org/licenses/by/4.0/).

Characteristic	Intervention Group (n=122)	Control Group (n=113)	P-value
Demographics			
Male sex, n (%)	87 (71)	90 (80)	0.2
Age, years	60 ± 16	64 ± 14	0.06
BMI, $kg.m^{-2}$	26 ± 5	26 ± 5	0.5
Coexisting Conditions, n (%)			
COPD	8 (7)	13 (12)	0.3
Hematologic neoplasm	7 (6)	1 (1)	0.07
Other	82 (67)	99 (88)	10^{-4}
None	33 (27)	13 (12)	0.003
Indication for ICU Admission, n (%)			
Septic shock	14 (11)	25 (22)	
Hemorrhagic shock	4 (3)	3 (3)	
Coma	1 (1)	4 (4)	
Trauma	5 (4)	0 (0)	0.05
Acute respiratory failure	33 (27)	31 (27)	
Elective surgery	12 (10)	10 (9)	
Emergent surgery	4 (3)	7 (6)	
Other	49 (40)	33 (29)	
Cause of ARDS, n (%)			
Pulmonary	81 (66)	80 (71)	0.5
Extrapulmonary	41 (34)	33 (29)	
Baseline Respiratory Variables			
PEEP, cmH_2O	10 ± 4	10 ± 4	0.7
Tidal volume, $mL.kg^{-1}$ PBW	6.5 ± 0.9	6.8 ± 1.3	0.02
Respiratory rate, per min	25 ± 4	24 ± 5	0.02
Pplat, cmH_2O	23 ± 5	24 ± 5	0.4
Static pulmonary compliance, $ml.cmH_2O^{-1}$	37 ± 15	37 ± 18	0.8
Driving pressure, cmH_2O	13 ± 5	13 ± 4	0.4
PaO_2, mmHg	82 ± 25	89 ± 32	0.1
PaO_2/FiO_2, mmHg	117 ± 39	117 ± 43	0.6
PaO_2/FiO_2 <100 mmHg, n (%)	45 (37)	46 (41)	0.5
$PaCO_2$ mmHg	45 ± 10	44 ± 10	0.5
FiO_2, %	74 ± 21	78 ± 20	0.1
Arterial pH	7.32 ± 0.10	7.31 ± 0.11	0.4
Serum bicarbonate, $mmol.L^{-1}$	23 ± 5	22 ±5	0.04
Baseline Hemodynamic Status			
Mean arterial blood pressure, mmHg	78.5 ± 14.5	78.1 ± 13.3	0.9
Heart rate, per min	99 ± 22	94 ± 24	0.06
Serum lactate, $mmol.L^{-1}$	2.39 ± 1.85	2.78 ± 3.02	0.9
Need for norepinephrine, n (%)	71 (58)	81 (72)	0.04

Baseline Renal Status			
Serum creatinine, µmol.L^{-1}	122 ± 86	146 ± 94	0.01
Need for renal replacement therapy, n (%)	7 (6)	7 (6)	0.6
Baseline Septic Status			
Under antibiotic therapy, n (%)	107 (88)	102 (90)	0.7
Abdominal sepsis, n (%)	18 (15)	22 (19)	0.4
Urinary tract infection, n (%)	3 (3)	2 (2)	1
Pneumonia, n (%)	73 (60)	68 (60)	1
Septicemia, n (%)	2 (2)	1 (1)	1
Soft tissue infection	1 (1)	0 (0)	1
Other infection	14 (11)	14 (12)	0.8
Corticosteroid therapy, n (%)	28 (23)	26 (23)	1
Serum bilirubin, µmol.L^{-1}	30 ± 49	23 ± 33	0.8
Baseline Severity of Illness			
SAPS II	51 ± 18	51 ± 16	1
SOFA	9 ± 4	10 ± 3	0.1
McCabe classification, n (%)			0.9
Category 1: Nonfatal disease	83 (69)	68 (62)	
Category 2: Ultimately fatal disease	33 (28)	38 (35)	
Category 3: Rapidly fatal disease	4 (3)	3 (3)	
Clinical Outcomes			
ICU mortality, n (%)	32 (26)	26 (23)	0.8
Death at day 90, n (%)	37 (30)	33 (29)	0.4
Plasma sRAGE, pg.mL^{-1} (median [interquartile])			
Baseline (day 0)	3,063 [1,773–5,180]	3,083 [1,730–4,910]	0.5
Day 1	1,974 [1,185–3,512]	2,048 [1,070–3,518]	0.9
Day 2	1,455 [830–2,584	1,607 [826–2,650]	0.6
Day 3	1,240 [757–2,046]	1,454 [653–2,619]	0.3
Day 4	1,139 [655–1,900]	1,472 [674–2,1695]	0.06
Day 6	1,089 [573–1,754]	1,237 [581–2,124]	0.3
Lung morphology, n (%)			
Focal ARDS	37 (30)	49 (43)	0.04
Nonfocal ARDS	87 (70)	64 (57)	0.03

Data are presented as mean ± standard deviation (SD) unless otherwise indicated. P-values were calculated for comparisons between patients randomized to the intervention group versus those randomized to the control group. Percentages may not exactly total 100% because of rounding. *The body mass index (BMI) is the weight in kilograms divided by the square of the height in meters. COPD: chronic obstructive pulmonary disease. ICU: intensive care unit. ARDS: acute respiratory distress syndrome. PEEP: positive end-expiratory pressure. Pplat: inspiratory plateau pressure.* PaO_2*: partial pressure of arterial oxygen.* FiO_2*: fraction of inspired oxygen. SAPS II: simplified acute physiology score II. SOFA: Sequential Organ Failure Assessment score. sRAGE: soluble receptor for advanced glycation end-products.*

Table S2. Baseline Characteristics and Clinical Outcomes of the LIVE Study Sample.

Characteristic	sRAGE measured (n=235)	sRAGE not measured (n=165)	P-value
Demographics			
Male sex, n (%)	177 (75)	124 (75)	0.9
Age, years	62 ± 15	62 ± 15	0.9
BMI, kg.m^{-2}	26 ± 5	26 ± 5	0.5
Coexisting Conditions, n (%)			
COPD	21 (9)	19 (12)	0.4
Hematologic neoplasm	8 (3)	8 (5)	0.5
Chronic dialysis	0 (0)	6 (4)	0.005
Other	181 (77)	87 (52)	$<10^{-4}$
None	46 (20)	55 (33)	0.002
Indication for ICU Admission, n (%)			
Septic shock	39 (17)	30 (18)	0.001
Hemorrhagic shock	7 (3)	5 (3)	
Coma	5 (2)	9 (5)	
Trauma	5 (2)	10 (6)	
Acute respiratory failure	64 (27)	68 (41)	
Elective surgery	22 (9)	7 (4)	
Emergent surgery	11 (5)	5 (3)	
Other	82 (35)	31 (19)	
Cause of ARDS, n (%)			
Pulmonary	161 (69)	131 (79)	0.02
Extrapulmonary	74 (31)	34 (21)	
Baseline Respiratory Variables			
PEEP, cmH_2O	10 ± 3	10 ± 3	0.1
Tidal volume, mL.kg^{-1} PBW	6.6 ± 1.3	6.6 ± 1.1	0.2
Respiratory rate, per min	24 ± 5	25 ± 6	0.5
Pplat, cmH_2O	23 ± 5	25 ± 6	0.01
Static pulmonary compliance, ml.cmH_2O^{-1}	37 ± 17	32 ± 23	10^{-4}
Driving pressure, cmH_2O	13 ± 5	15 ± 6	$<10^{-4}$
PaO_2, mmHg	86 ± 29	85 ± 28	0.9
PaO_2/FiO_2, mmHg	117 ± 41	115 ± 43	0.7
PaO_2/FiO_2 <100 mmHg, n (%)	91 (37)	66 (40)	0.6
$PaCO_2$ mmHg	45 ± 10	48 ± 12	0.02
FiO_2, %	76 ± 20	78 ± 20	0.5
Arterial pH	7.32 ± 0.11	7.31 ± 0.10	0.6
Serum bicarbonate, mmol.L^{-1}	23 ± 5	23 ± 5	0.6
Baseline Hemodynamic Status			
Mean arterial blood pressure, mmHg	78.3 ± 13.9	80.4 ± 15.2	0.1
Heart rate, per min	96 ± 23	103 ± 23	0.02
Serum lactate, mmol.L^{-1}	2.6 ± 3.0	2.7 ± 2.9	0.9
Need for norepinephrine, n (%)	152 (65)	94 (57)	0.1
Baseline Renal Status			
Serum creatinine, µmol.L^{-1}	133 ± 91	151 ± 162	0.9
Need for renal replacement therapy, n (%)	14 (6)	12 (7)	0.6
Baseline Septic Status			
Under antibiotic therapy, n (%)	209 (89)	151 (93)	0.2
Abdominal sepsis, n (%)	40 (17)	21 (12)	0.3
Urinary tract infection, n (%)	5 (2)	6 (4)	0.4

Pneumonia, n (%)	141 (60)	116 (70)	0.02
Septicemia, n (%)	3 (1)	0 (0)	0.2
Soft tissue infection	1 (0.4)	2 (1)	0.6
Other infection	28 (12)	12 (7)	0.17
Corticosteroid therapy, n (%)	54 (23)	37 (23)	0.9
Serum bilirubin, µmol.L^{-1}	26 ± 42	25 ± 44	0.5
Baseline Severity of Illness			
SAPS II	51 ± 17	52 ± 16	0.5
SOFA	10 ± 4	9 ± 4	0.06
McCabe classification, n (%)			0.2
Category 1: Nonfatal disease	151 (66)	112 (71)	
Category 2: Ultimately fatal disease	71 (31)	38 (24)	
Category 3: Rapidly fatal disease	7 (3)	8 (5)	
Clinical Outcomes			
ICU mortality, n (%)	58 (25)	32 (20)	0.2
Death at day 90, n (%)	70 (33)	39 (28)	0.3
Lung morphology, n (%)			
Focal ARDS	47 (28)	86 (37)	0.1
Nonfocal ARDS	118 (72)	149 (63)	
Randomization group, n (%)			
Control	82 (50)	122 (52)	0.7
Intervention	83 (50)	113 (48)	

Data are presented as mean ± standard deviation (SD) unless otherwise indicated. P-values were calculated for comparisons between patients with sRAGE measurements available at baseline and those without sRAGE measurements available at baseline. Percentages may not exactly total 100% because of rounding. *The body mass index (BMI) is the weight in kilograms divided by the square of the height in meters. sRAGE: soluble receptor for advanced glycation end-products. COPD: chronic obstructive pulmonary disease. ICU: intensive care unit. ARDS: acute respiratory distress syndrome. PEEP: positive end-expiratory pressure. Pplat: inspiratory plateau pressure. PaO_2: partial pressure of arterial oxygen. FiO_2: fraction of inspired oxygen. SAPS II: simplified acute physiology score II. SOFA: Sequential Organ Failure Assessment score.*

Table S3. Multivariable Marginal Cox Survival Sensitivity Analyses of Death at Day 90, Considering Plasma sRAGE both at Baseline and as a Time-varying Covariate, When Imputing Missing Data Using LOCF was Used to Impute Missing Data.

	**Hazard Ratio [95% CI]**	**P**
**Baseline plasma sRAGE***	1.52 [1.16 - 2.00]	0.002
**Increase in plasma sRAGE****	1.01 [1.01 - 1.02]	$<10^{-3}$
**Baseline plasma sRAGE***	1.45 [1.15 - 1.83]	0.002
**Increase in plasma sRAGE****	1.01 [1.01 - 1.02]	0.003
Age — yr	1.02 [0.99 - 1.05]	0.09
SAPS II	1.03 [1.01 - 1.05]	0.01
McCabe category 2	1.69 [0.88 - 3.24]	0.1
McCabe category 3	1.15 [0.35 - 3.78]	0.8
History of hematologic cancer	0.64 [0.16 - 2.56]	0.5
History of solid cancer	5.44 [2.47 - 11.96]	$<10^{-3}$
Shock at baseline	1.33 [0.64 - 2.78]	0.4
Need for continuous renal replacement therapy at baseline	1.56 [0.70 - 3.49]	0.3
Corticosteroid therapy at baseline	0.98 [0.51 - 1.89]	0.9
Randomization to the personalized ventilation group	1.04 [0.57 - 1.93]	0.9
Focal lung morphology (after post-hoc reclassification)	0.91 [0.47 - 1.77]	0.8
Correct classification of lung morphology at baseline	0.30 [0.15 - 0.60]	0.001

*Hazard Ratio is expressed for each one-log increment in baseline plasma sRAGE. **Hazard Ratio is expressed for each one-log increase in plasma sRAGE per unit of time. *n=1,272 repeated sRAGE measures from 235 patients available for complete-case multivariable analysis. SAPS II: simplified acute physiology score II. sRAGE: soluble receptor for advanced glycation end-products. LOCF: last observation carried forward.*

Table S4. Multivariable Marginal Cox Survival Sensitivity Analyses of Death at Day 90, Considering Plasma sRAGE both at Baseline and as a Time-varying Covariate, When Restricting Analyses to Plasma sRAGE values from days 0, 1, 2, and 3 in Patients who Survived to Day 3.

	**Hazard Ratio [95% CI]**	**P**
**Baseline plasma sRAGE***	1.52 [1.01 - 2.28]	0.045
**Increase in plasma sRAGE****	1.01 [1.01 - 1.02]	0.006
**Baseline plasma sRAGE***	1.58 [1.09 - 2.28]	0.016
**Increase in plasma sRAGE****	1.01 [1.01 - 1.02]	0.017
Age — yr	1.03 [0.99 - 1.06]	0.05
SAPS II	1.02 [0.99 - 1.04]	0.1
McCabe category 2	1.78 [0.81 - 3.95]	0.2
McCabe category 3	1.57 [0.43 - 5.72]	0.5
History of hematologic cancer	0.94 [0.20 - 4.45]	0.9
History of solid cancer	9.03 [3.06 - 26.67]	$<10^{-3}$
Shock at baseline	1.33 [0.57 - 3.11]	0.5
Need for continuous renal replacement therapy at baseline	1.96 [0.78 - 4.90]	0.2
Corticosteroid therapy at baseline	0.98 [0.46 - 2.12]	0.9
Randomization to the personalized ventilation group	1.01 [0.48 - 2.12]	0.9
Focal lung morphology (after post-hoc reclassification)	0.82 [0.38 - 1.73]	0.6
Correct classification of lung morphology at baseline	0.32 [0.14 - 0.71]	0.005

*Hazard Ratio is expressed for each one-log increment in baseline plasma sRAGE. **Hazard Ratio is expressed for each one-log increase in plasma sRAGE per unit of time. *n=773 repeated sRAGE measures from 204 patients available for complete-case multivariable analysis. SAPS II: simplified acute physiology score II. sRAGE: soluble receptor for advanced glycation end-products.*

Table S5. Baseline Characteristics and Clinical Outcomes of Patients with Focal and Nonfocal ARDS.

Characteristic	Focal ARDS (n=86)	Nonfocal ARDS (n=149)	P-value
Demographics			
Male sex, n (%)	70 (81)	107 (72)	0.1
Age, years	60 ± 14	63 ± 15	0.2
BMI, $kg.m^{-2}$	27 ± 4	26 ± 5	0.01
Coexisting Conditions, n (%)			
COPD	9 (10)	12 (8)	0.6
Hematologic neoplasm	1 (1)	7 (5)	0.2
Chronic dialysis	0 (0)	0 (0)	1
Other	63 (73)	118 (80)	0.3
None	21 (24)	25 (17)	0.1
Indication for ICU Admission, n (%)			
Septic shock	21 (24)	18 (12)	
Hemorrhagic shock	2 (2)	5 (3)	
Coma	2 (2)	3 (2)	
Trauma	2 (2)	3 (2)	0.09
Acute respiratory failure	23 (27)	41 (27)	
Elective surgery	5 (6)	17 (11)	
Emergent surgery	7 (8)	4 (3)	
Other	24 (28)	58 (39)	
Cause of ARDS, n (%)			
Pulmonary	52 (60)	109 (73)	0.06
Extrapulmonary	34 (40)	40 (27)	
Baseline Respiratory Variables			
PEEP, cmH_2O	10 ± 4	11 ± 4	0.07
Tidal volume, $mL.kg^{-1}$ PBW	6.6 ± 1.2	6.7 ± 1.1	0.4
Respiratory rate, per min	24 ± 5	25 ± 6	0.3
Pplat, cmH_2O	23 ± 5	23 ± 5	0.7
Static pulmonary compliance, $ml.cmH_2O^{-1}$	37 ± 13	37 ± 18	0.3
Driving pressure, cmH_2O	13 ± 4	13 ± 5	0.7
PaO_2, mmHg	86 ± 28	86 ± 29	0.9
PaO_2/FiO_2, mmHg	116 ± 39	118 ± 42	0.9
PaO_2/FiO_2 <100 mmHg, n (%)	31 (36)	60 (40)	0.5
$PaCO_2$ mmHg	43 ± 9	45 ± 11	0.2
FiO_2, %	76 ± 20	76 ± 20	0.7
Arterial pH	7.32 ± 0.10	7.32 ± 0.11	0.9
Serum bicarbonate, $mmol.L^{-1}$	22 ± 5	23 ±5	0.1
Baseline Hemodynamic Status			
Mean arterial blood pressure, mmHg	79.2 ± 15.8	77.8 ± 12.8	0.9
Heart rate, per min	97 ± 24	96 ± 22	0.7
Serum lactate, $mmol.L^{-1}$	2.29 ± 1.66	2.75 ± 3.59	0.8
Need for norepinephrine, n (%)	52 (61)	100 (67)	0.3
Baseline Renal Status			
Serum creatinine, $\mu mol.L^{-1}$	134 ± 91	151 ± 162	0.9
Need for renal replacement therapy, n (%)	6 (7)	8 (5)	0.6
Baseline Septic Status			
Under antibiotic therapy, n (%)	71 (83)	138 (93)	0.02
Abdominal sepsis, n (%)	17 (20)	23 (15)	0.4
Urinary tract infection, n (%)	0 (0)	5 (3)	0.1

Pneumonia, n (%)	43 (50)	98 (66)	0.02
Septicemia, n (%)	2 (2)	1 (1)	0.6
Soft tissue infection	10 (0)	1 (1)	1
Other infection	13 (15)	15 (10)	0.3
Corticosteroid therapy, n (%)	17 (20)	37 (25)	0.4
Serum bilirubin, µmol.L^{-1}	26 ± 42	25 ± 44	0.5
Baseline Severity of Illness			
SAPS II	51 ± 17	51 ± 16	
SOFA	10 ± 4	10 ± 4	1
McCabe classification, n (%)			0.8
Category 1: Nonfatal disease	56 (67)	95 (65)	0.9
Category 2: Ultimately fatal disease	25 (30)	46 (32)	
Category 3: Rapidly fatal disease	2 (2)	5 (3)	
Clinical Outcomes			
ICU mortality, n (%)	20 (23)	38 (25)	0.8
Death at day 90, n (%)	23 (30)	47 (36)	0.4
Plasma sRAGE, pg.mL^{-1} (median [interquartile])			
Baseline (day 0)	2,346 [1,133–3,218]	3,577 [2,113–8,480]	0.0001
Day 1	1,451 [853–3,413]	2,255 [1,393–3,967]	0.0008
Day 2	1,330 [813–2,584]	1,548 [839–2,700]	0.5
Day 3	1,486 [688–1,981]	1,271 [705–2,376]	0.9
Day 4	1,214 [553–2,353]	1,261 [670–2,136]	0.7
Day 6	1,230 [675–2,084]	1,090 [547–1,757]	0.2
Randomization group, n (%)			
Control	37 (43)	85 (57)	0.04
Intervention	49 (57)	64 (43)	

Data are presented as mean ± standard deviation (SD) unless otherwise indicated. P-values were calculated for comparisons between patients with focal ARDS and those with nonfocal ARDS. Percentages may not exactly total 100% because of rounding. *The body mass index (BMI) is the weight in kilograms divided by the square of the height in meters. COPD: chronic obstructive pulmonary disease. ICU: intensive care unit. ARDS: acute respiratory distress syndrome. PEEP: positive end-expiratory pressure. Pplat: inspiratory plateau pressure. PaO$_2$: partial pressure of arterial oxygen. FiO$_2$: fraction of inspired oxygen. SAPS II: simplified acute physiology score II. SOFA: Sequential Organ Failure Assessment score. sRAGE: soluble receptor for advanced glycation end-products.*

Table S6. Effects of Time, Group, and Time x Group Interaction on Plasma sRAGE Measured at Study Timepoints, Stratified by Focal vs. Nonfocal Lung Morphology and Matched vs. Mismatched Personalized Ventilation Strategy.

Lung Morphology	Classification of Lung Morphology at Baseline	Regression Coefficient for Group Effect [95% CI]	Timepoint	Regression Coefficient for Time Effect [95% CI]	Regression Coefficient for Time x Group Interaction [95% CI]
Focal	*Correctly classified*	530 [-2,803; 3,862] (P=0.8)	*Day 1*	2,452 [-2,101–7,006] (P = 0.3)	-1,734 [-4,525–1057] (P = 0.2)
			Day 2	5,320 [658–9,982] (P = 0.03)	**-3,441 [-6,310– -572] (P = 0.02)**
			Day 3	5,664 [868–10,460] (P = 0.02)	**-3,518 [-6,434– -602] (P = 0.02)**
			Day 4	1,831 [-2,996–6,659] (P = 0.5)	-1,601 [-4,565–1,363] (P = 0.3)
			Day 6	2,188 [-2,721–7,096] (P = 0.4)	-1,999 [-5,039–1,040] (P = 0.2)
	Incorrectly classified	-4,02 [-7,045; -999] (P=0.01)	*Day 1*	-7,690 [-13,214– -2,167] (P = 0.02)	**3,663 [566–6,759] (P = 0.02)**
			Day 2	-9,518 [-15,093– -3,943] (P = 0.001)	**4,737 [1,549–7,925] (P = 0.004)**
			Day 3	-9,142 [-14,756– -3,528] (P = 0.001)	**4,106 [851–7,362] (P = 0.02)**
			Day 4	-9,361 [-15031– -3,692] (P = 0.01)	**4,207 [857–7,557] (P = 0.01)**
			Day 6	-9,513 [-15,271– -3755] (P = 0.001)	**4,743 [1,245–8,241] (P = 0.008)**
Nonfocal	*Correctly classified*	-1,209 [-3,266; 849] (P=0.3)	*Day 1*	**-4,545 [-8,165– -925] (P = 0.01)**	567 [-1,748–2,862] (P = 0.6)
			Day 2	**-7,114 [-10,735– -3,493] ($P<10^{-4}$)**	1,095 [-1,205–3,395] (P = 0.4)
			Day 3	**-7,795 [-11,417– -4,174] ($P<10^{-4}$)**	1,354 [-970–3678] (P = 0.3)
			Day 4	**-8,005 [-11,768– -4,243] ($P<10^{-4}$)**	1,353 [-1,020–3,726] (P = 0.3)
			Day 6	**-7,767 [-11,627– -3,906] ($P<10^{-4}$)**	871 [-1,597–3,339] (P = 0.5)
	Incorrectly classified	-1,876 [-6,537; 2,785] (P=0.4)	*Day 1*	-764 [-876–7,234] (P = 0.9)	-854 [-6,467–4,760] (P = 0.8)
			Day 2	-6,274 [-14,335–1,786] (P = 0.1)	1,227 [-4,475–6,929] (P = 0.7)
			Day 3	**-9,828 [-17,952– -1,704] (P = 0.02)**	3,383 [-2,341–9,108] (P = 0.2)
			Day 4	**-9,277 [-17,676– -879] (P = 0.03)**	2,713 [-3,179; 8,604] (P = 0.4)
			Day 6	-8,971 [-17,788– -154] (P = 0.05)	2,521 [-3,694–8736] (P = 0.4)

sRAGE: soluble receptor for advanced glycation end-products.

REFERENCES

1. Constantin, J.-M.; Jabaudon, M.; Lefrant, J.-Y.; Jaber, S.; Quenot, J.-P.; Langeron, O.; Ferrandière, M.; Grelon, F.; Seguin, P.; Ichai, C.; et al. Personalised Mechanical Ventilation Tailored to Lung Morphology versus Low Positive End-Expiratory Pressure for Patients with Acute Respiratory Distress Syndrome in France (the LIVE Study): A Multicentre, Single-Blind, Randomised Controlled Trial. *Lancet Respir Med* **2019**, *7*, 870–880.
2. Mrozek, S.; Jabaudon, M.; Jaber, S.; Paugam-Burtz, C.; Lefrant, J.-Y.; Rouby, J.-J.; Asehnoune, K.; Allaouchiche, B.; Baldesi, O.; Leone, M.; et al. Elevated Plasma Levels of sRAGE Are Associated With Nonfocal CT-Based Lung Imaging in Patients With ARDS: A Prospective Multicenter Study. *Chest* **2016**, *150*, 998–1007.
3. Jabaudon, M.; Godet, T.; Futier, E.; Bazin, J.-É.; Sapin, V.; Roszyk, L.; Pereira, B.; Constantin, J.-M.; AZUREA group. Rationale, Study Design and Analysis Plan of the Lung Imaging Morphology for Ventilator Settings in Acute Respiratory Distress Syndrome Study (LIVE Study): Study Protocol for a Randomised Controlled Trial. *Anaesth Crit Care Pain Med* **2017**, *36*, 301–306.
4. Brower, R.G.; Lanken, P.N.; MacIntyre, N.; Matthay, M.A.; Morris, A.; Ancukiewicz, M.; Schoenfeld, D.; Thompson, B.T.; National Heart, Lung, and Blood Institute ARDS Clinical Trials Network Higher versus Lower Positive End-Expiratory Pressures in Patients with the Acute Respiratory Distress Syndrome. *N. Engl. J. Med.* **2004**, *351*, 327–336.
5. Mercat, A.; Richard, J.-C.M.; Vielle, B.; Jaber, S.; Osman, D.; Diehl, J.-L.; Lefrant, J.-Y.; Prat, G.; Richecoeur, J.; Nieszkowska, A.; et al. Positive End-Expiratory Pressure Setting in Adults with Acute Lung Injury and Acute Respiratory Distress Syndrome: A Randomized Controlled Trial. *JAMA* **2008**, *299*, 646–655.
6. Le Gall, J.R.; Lemeshow, S.; Saulnier, F. A New Simplified Acute Physiology Score (SAPS II) Based on a European/North American Multicenter Study. *JAMA* **1993**, *270*, 2957–2963.
7. McCabe, W.R.; Jackson, G.G. Gram-Negative Bacteremia: I. Etiology and Ecology. *Arch. Intern. Med.* **1962**, *110*, 847–855.
8. Faraone, S.V. Interpreting Estimates of Treatment Effects: Implications for Managed Care. *P T* **2008**, *33*, 700–711.
